# The Health of the British Army

**Published:** 1905-08-12

**Authors:** 


### THE HEALTH OF THE BRITISH ARMY.

From the report of the Army Medical Department for the year 1903, which was recently published, it appears that the average strength of European troops serving at home and abroad in 1903 was 242,182 warrant officers, non-commissioned officers, and men. The total number of admissions to hospital was 183,598, the total deaths, 1,881, and the total number of men finally discharged from the service on account of disease and injuries was 4,922. The admissions represent a ratio of 758·1 per 1,000 of strength as above; the deaths one of 7·13 per 1,000 on a strength of 263,888, which includes men not shown in the returns received. In 1902 the admission ratio was 751·9 and the death-rate 7·52 per 1,000, while for the decennial period from 1893 to 1902 the corresponding ratios were 927·4 and 8·57 respectively. The principal causes of admission during the year were venereal affections, diseases of the digestive system, malarial fevers, injuries, diseases of the skin, and diseases of the respiratory system. The chief causes of death were enteric (481), injuries (290), diseases of the digestive system (205), diseases of the respiratory system, excluding tubercle of the lung (203), and diseases of the circulatory system (142). The invaliding records show that diseases of the circulatory system (942), diseases of the nervous system (668), diseases of the digestive system (507), tubercular diseases (338), and diseases of the organs of locomotion (311), head the list in the case of men finally discharged as unfit for further service.

In the United Kingdom there was a slight increase in the admission rate for venereal disease during the year 1903, but it is still below the average rate for the preceding 10 years. At Gibraltar there was an increase in the admission rate for venereal diseases, which, however, was lower than the average for the decennial period. At Malta venereal disease showed an increased admission rate as compared with 1902, but it was still below the average rate for the preceding 10 years. In Canada the health of the troops was very satisfactory. There was a large decrease in the number of cases of venereal disease. Enteric fever was again largely prevalent in Bermuda, but the admission rate was considerably lower than in the previous year. There was an increase in the venereal admission rates, but it is still below the decennial average.

In the Barbadoes Command (in which is included the health statistics of the troops of St. Lucia and at Trinidad), there was a large decrease in the prevalence of malarial fevers as compared with the previous year, which may be attributed to various causes, amongst which were the more healthy character of the season of 1903; the departure of troops who had been several years in the station and had suffered from malaria, and their relief by healthy men from a non-malarious station; and also to the large amount of draining and clearing accomplished at St. Lucia during the year, and to the great diminution in the number of anopheles brought about by these and other anti-malarial measures. Venereal diseases also showed a large decrease as compared with 1902.

In the South African Command, with an average strength of 27,680, the admission, deaths, and constantly sick rates were 797·8, 11·06, and 60·03 per 1,000 respectively. In order of frequency the most important causes of admission were diseases of the digestive system, injuries, venereal diseases, diseases of the skin, enteric fever, influenza, diseases of the respiratory system, dysentery, and rheumatism. Enteric prevalence usually begins in October, and declines about May. As in India, so is the teaching of South African experience that the key to the prevention of this disease is the prevention of soil pollution. Polluting the soil of camps or barracks, or of their surroundings, should be regarded as a very grave offence, and perfection should be aimed at in connection with latrine arrangements, and in regard to the methods used for the removal and disposal of the excreta and refuse. In proportion as the ideal is approached in these matters, so will the chances of enteric fever making its appearance be lessened. In Ceylon the health of the troops was good. In India there was a decrease in the admission, mortality, constantly sick and invaliding rates. The chief causes of sickness, as usual, were malarial fevers and venereal diseases, which together caused 47·74 per cent. of the admissions for all diseases. In Egypt the admission and constantly sick rates were higher and the mortality rate lower than 1902, and all were lower than the decennial average rates.

